# How the Italian NHS Is Fighting Against the COVID-19 Emergency

**DOI:** 10.3389/fpubh.2020.00167

**Published:** 2020-05-08

**Authors:** Stefania Boccia, Fidelia Cascini, Martin McKee, Walter Ricciardi

**Affiliations:** ^1^Section of Hygiene, University Department of Health Sciences and Public Health, Università Cattolica del Sacro Cuore, Rome, Italy; ^2^Department of Woman and Child Health and Public Health - Public Health Area, Fondazione Policlinico Universitario A. Gemelli IRCCS, Rome, Italy; ^3^London School of Hygiene and Tropical Medicine, London, United Kingdom

**Keywords:** Covid-19, Italy, pandemic, healthcare systems, emergency

Italy, with more than 183,957 cases as of April 22nd (1) has the second highest burden of coronavirus disease 2019 (COVID-19) in Europe after Spain, and the third highest worldwide. The speed with which the epidemic grew took all concerned by surprise (2). Within a week of the first case being identified in Codogno, Lombardy, the number had grown to 821, with 21 deaths. This placed the local health services under exceptional pressure and, as in Spain (3), created tensions within the decentralized Italian health system.

Italy comprises 20 regions, with differing levels of autonomy. The Italian Prime Minister threatened to take back powers from the regions and autonomous provinces as they were “in charge of implementing healthcare but not prepared to face a national emergency” (4). The national response came in the form of a series of seven Decrees from the Presidency of the Council of Ministers (in effect the Prime Minister's office) progressively extending countermeasures.

After the first declaration of emergency of January 31st, a Decree (February 23rd) isolated cities with COVID-19 clusters within the northern Italian regions (Lombardy and Veneto) (Table 1). The following Deecrees adopted further restrictions, closing schools and universities, prohibiting all public events, such as concerts and major sports competitions, and limiting business hours. The last three Decrees imposed restrictions on mobility of the population. Early on, several towns had introduced varying forms of quarantine, but further clusters continued to emerge. As a consequence, the new Decrees extended restrictions from the Region of Lombardy to all of northern Italy and, by March 11th, to the entire country (Figure 1).

**Table 1:** The main Decrees in Italy during the COVID-19 pandemic.

31st Jan 2020	The Government declares the state of emergency
23rd – 25th Feb 2020	First containment measures in some municipalities of Lombardy, Veneto, Emilia-Romagna and Marche
1st Mar 2020	Lockdown for 11 municipalities in Lombardy and Veneto, and additional limitations for Emilia Romagna, Lombardy and Veneto
4th Mar 2020	Suspension of teaching activities lessons in schools/universities in the Country
9th Mar 2020	The Government allocates 845 millions to face the emergency. The lockdown is extended to Lombardy and other 14 provinces in Veneto, Emilia-Romagna and Marche
11th Mar 2020	Lockdown is extended to the Country
22nd Mar 2020	Suspension to the entire productive chain (unless “essential”, e.g. food production and distribution) in the Country

**Figure 1:**
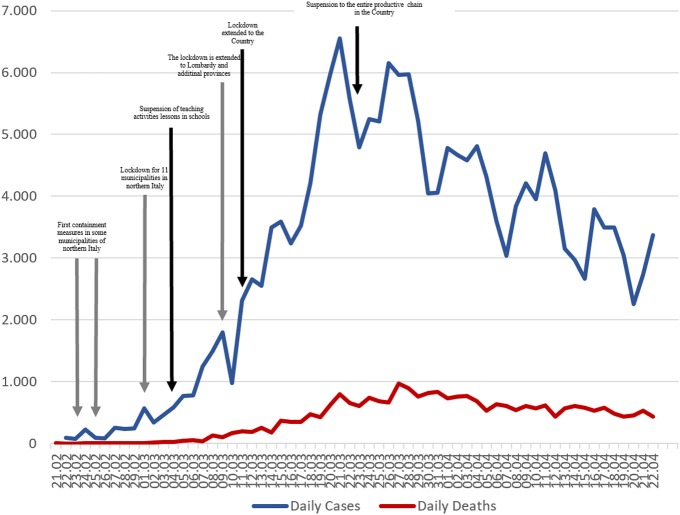
Number of new cases, deaths and total cases due to COVID-19 in Italy, from 21st February to 22nd April 2020. Gray arrows represent the Legislative Decrees with a regional impact. Black once the Legislative Decrees with national impact.

Detailed surveillance is being conducted by a Task Force in the Department of Infectious Diseases of the Instituto Superiore di Sanità (5). According to the latest available reports, three-quarters (70.8%) of cases were over 50 years of age and only 1.6% were aged 18 years or younger, with 27.4% between 19 and 50 years old. The majority (52.4%) were men, the same as in early reports from China (6). Healthcare workers represented 10.3% of the reported cases, and among them lethality was 0.3%.

As of April 22nd, nearly half of all cases were diagnosed in Lombardy (69,092), followed by Emilia Romagna (23,434 cases) and Piemonte (22,739 cases) (Supplementary Table S1). With clinical data available for 52,577 cases, most (35.7%) were classified as having mild pneumonia but 17.4% were severe (dyspnoea, respiratory rate ≥30/min, blood oxygen saturation ≤93%), and 1.9% were critical (respiratory failure, septic shock, and/or multiple organ dysfunction or failure), while 30% had few or no symptoms.

Obviously, international comparisons of case fatality must be interpreted with caution due to differences in the intensity of testing and, with deaths, the criteria for establishing the underlying cause. At present it appears that all deaths in someone who has tested positive for COVID-19 are attributed to the virus and this may, and probably is not the case everywhere. By April, 22nd, where we had 23,085 deaths, giving a case fatality rate of 12.3%. This is higher than has been reported in many other countries but is likely to be explained, at least in part, by the age distribution.

The median age of cases in Italy is 62 years, compared to 47 in China (6). However, the median age of those dying in Italy is 80 years. Again, noting the need for caution because of issues with denominators, there was a clear association between age and outcome. There were no deaths among those aged under 30 years old, but the case fatality rate was 19.1% in those aged 70 to 79, increasing to 27.1% in those 80 years and older. Outcomes were also strongly associated underlying conditions: 48.6% of deaths were among people with 3 or more comorbidities, 26.6% had two, 23.5% had one, and only 6 deaths (1.2%) were of people who had apparently been healthy.

The challenge to the National Health Service has been immense starting from the red zones in the Northern Italy. For instance, before the current crisis Lombardy had approximately 720 intensive care beds (2.9% of all hospital beds in the region) (7). In the first 18 days of the epidemic, 482 of them were required to treat patients with COVID-19 (7). In these circumstances the National Health Service has had to innovate. First, separate testing sites were established, and the Ministry of Health asked general practitioners to refer anyone meeting certain criteria based on their symptoms, to divert them from health facilities facing extreme pressure. Second, the Ministry of Health put in place measures to recruit additional doctors and nurses to increase the capacity of intensive care units (ICU). This included an extraordinary plan, launched on March 7th, to employ medical students and retired healthcare professionals. Meanwhile, on March 8th, €845 million was allocated for additional medical devices and equipment (8). Unfortunately, these measures have been implemented against a backdrop of the loss of many health care workers who have been quarantined or fallen ill with the infection, some of whom, tragically, have died.

The approaches taken by the Italian health system to the COVID-19 emergency have varied among the most severely affected regions fall into three broad types (9). Type 1 is a hospital based model, adopted in Lombardy. Type 2 is a territorial based model, in Veneto. Type 3 is a combined hospital-territorial model, as in Emilia-Romagna and Piedmont. The first type places the main emphasis on the role of hospitals, with a relatively low level of community testing. This has, as might be expected, been associated with substantial pressure on hospitals and, particularly, ICU beds. An average of 50% of those diagnosed with COVID-19 have been admitted to hospital in Lombardy (vs. an average of 45% in other regions). Although this seems a small difference, the duration of stay in ICUs means that, at any one time, he ratio of patients treated in ICUs to those treated at home is twice as high in Lombardy than in Veneto, Emilia Romagna and Piedmont. This also means that daily occupancy of ICU beds has been exceeding 100%, in contrast to Emilia-Romagna, the second most severely affected region, where occupancy is 38% (9).

The territorial management approach is characterized by a lower hospitalization rate and a higher incidence of testing. An extreme example is the town of Vò, in Veneto region, where all 3,000 inhabitants were tested (10). In Veneto, only 22% of patients with a positive result are hospitalized (compared to the 45–50% of the other Italian regions) and nasopharyngeal swabs, which are also administered to asymptomatic individuals, reached 3.13% of the regional population (vs. an average of 1.25% of the other regions) (9). The combined hospital-territorial management model, adopted in Emilia-Romagna and Piedmont, is characterized by an intermediate level of hospitalization and an intermediate level of testing.

In a situation such as the current pandemic, where the optimal course of action is uncertain, Italy's decentralized structure has provided an important natural experiment. While there is still much to be learned, the emerging evidence points to the territorial management model being the best response to this emergency.

## Author Contributions

SB, WR, and FC: Substantial contributions to the conception and design of the work. MM: Critical revision of the manuscript, interpretation of data for the work.

## Conflict of Interest

The authors declare that the research was conducted in the absence of any commercial or financial relationships that could be construed as a potential conflict of interest.
